# Anxiety, prenatal distress, and resilience during the first trimester of gestation

**DOI:** 10.1590/1980-220X-REEUSP-2023-0290en

**Published:** 2024-05-13

**Authors:** Cristina Liébana-Presa, Rubén García-Fernández, Cristian Martín-Vázquez, María Cristina Martínez-Fernández, Pedro Hidalgo-Lopezosa

**Affiliations:** 1 Universidad de León, Facultad de Ciencias de la Salud, Departamento de Enferemría y Fisioterapia, Ponferrada, España.; 2 Universidad de Córdoba, Facultad de Medicina y Enfermería, Departamneto de Enfermeira, Farmacología y Fisioterpia, Córdoba, España.

**Keywords:** Pregnancy, Stress, Psychological, Anxiety, Resilience, Psychological, Gravidez, Estresse Psicológico, Ansiedade, Resiliência Psicológica, Embarazo, Estrés Psicológico, Ansiedad, Resiliencia Psicológica

## Abstract

**Objective:**

To describe and analyze the relationship between pregnancy-related anxiety, prenatal distress, and individual resilience in pregnant women during the first trimester of pregnancy and compare it with the obstetric variable of parity.

**Method:**

Quantitative, descriptive, cross-sectional study using non-probabilistic circumstantial sampling. A total of 144 women participated. The Prenatal Distress Questionnaire, the Resilience Scale, and the Pregnancy-Related Anxiety Questionnaire were used. A descriptive analysis with measures of central tendency was performed, and the reliability of the instruments was assessed.

**Results:**

The average age was 33.57 years. 58.3% were multiparous and 41.7% primiparous. Anxiety was found in 21.5% and very high levels of resilience in 54.9%. Primiparous women showed higher levels of worry about the future and fear of childbirth than multiparous women. Pregnant women with high resilience showed lower levels of anxiety and stress.

**Conclusion:**

Pregnant women with higher levels of resilience show less anxiety and stress during the first trimester of pregnancy. Primiparous women show more anxiety and stress than multiparous women.

## RESUMEN

**Objetivo:**

Describir y analizar la relación entre la ansiedad relacionada con el embarazo, el distrés prenatal y la resiliencia individual, en mujeres gestantes durante el primer trimestre de embarazo y compararlo con la variable obstétrica de paridad.

**Método:**

Estudio cuantitativo, descriptivo y transversal utilizando muestreo no probabilístico circunstancial. Participaron 144 mujeres. Se emplearon el Cuestionario de Distrés Prenatal, la Escala de Resiliencia y el Cuestionario de Ansiedad Relacionada con el Embarazo. Se realizó un análisis descriptivo con medidas de tendencia central y se evaluó la confiabilidad de los instrumentos.

**Resultados:**

La edad promedio fue de 33,57 años. El 58,3% eran multíparas y el 41,7% primíparas. El 21,5% presenta ansiedad y el 54,9% niveles muy altos de resiliencia. Las primíparas mostraron mayores niveles preocupación sobre el futuro y miedo al parto que las multíparas. Las gestantes con alta resiliencia muestran valores más bajos en ansiedad y estrés.

**Conclusiones:**

Las gestantes con niveles más elevados de resiliencia manifiestan menos ansiedad y estrés durante el primer trimestre de embarazo. Las primíparas presentan más ansiedad y estrés que las multíparas.

## RESUMO

**Objetivo:**

Descrever e analisar a relação entre a ansiedade relacionada à gravidez, o estresse pré-natal e a resiliência individual em mulheres grávidas durante o primeiro trimestre da gravidez e compará-la com a variável obstétrica da paridade.

**Método:**

Estudo quantitativo, descritivo, transversal usando amostragem circunstancial não probabilística. Um total de 144 mulheres participaram. Foram utilizados o Questionário de Estresse Pré-natal, a Escala de Resiliência e o Questionário de Ansiedade Relacionada à Gravidez. Foi realizada uma análise descritiva com medidas de tendência central, e a confiabilidade dos instrumentos foi avaliada.

**Resultados:**

A idade média foi de 33,57 anos. 58,3% eram multiparas e 41,7% primíparas. Ansiedade foi encontrada em 21,5% e níveis muito altos de resiliência em 54,9%. Mulheres primíparas apresentaram níveis mais altos de preocupação com o futuro e medo do parto do que mulheres multiparas. Mulheres grávidas com alta resiliência mostraram níveis mais baixos de ansiedade e estresse.

**Conclusões:**

Mulheres grávidas com níveis mais altos de resiliência apresentam menos ansiedade e estresse durante o primeiro trimestre da gravidez. Mulheres primíparas mostram mais ansiedade e estresse do que mulheres multiparas.

## INTRODUCTION

Gestation is a transitional stage involving significant physical, emotional, and social changes^(1,2)^ that may pose a risk to the health of both the woman and the newborn. Scientific evidence shows that anxiety and distress significantly affect perinatal health, and that resilience can help to reduce the levels of these two variables, facilitating positive adaptation to stressful situations and acting proactively to identify and prevent potential problems: in short, to remain in good health during pregnancy^(3,4)^.

Anxiety is a common emotion that affects both the general population and pregnant women^(5,6)^, with anxiety during pregnancy affecting one in five women(7). During pregnancy, anxiety related to concerns about the current pregnancy, future birth or fetal development may also arise, which is termed pregnancy-specific anxiety(8). Despite its global prevalence, anxiety varies significantly between countries, due to cultural influences. Even in Europe, where multiculturalism is high, the prevalence varies from 7.7% to 36.5%(9), with anxiety levels of up to 44.5% being recorded in Spain during the first trimester(10).

Stress is another symptom frequently experienced by pregnant women. Previous research has conceptualized stress during pregnancy in a practical way as the number of severe life events or daily difficulties that occur while a woman is pregnant^(11,12)^. Pregnant women, especially during the first trimester of pregnancy, have been reported to suffer from pregnancy-specific stress(13), which refers to concerns about fetal health, childbirth, or the onset of pregnancy-related physical problems(14).

Resilience is a positive psychological resource which can prevent mental disorders, and is a dynamic process that enables people at any stage of life to cope with adversity, bounce back after difficulties, manage unpleasant feelings, and adapt to change^(5,15)^. Resilience appears to work as a protective factor against prenatal anxiety, ensuring it happens less often or decreasing its negative effects^(5,16)^. Resilience also gives pregnant women a feeling of coherence, which helps them cope with anxiety and fear of childbirth(16), and enables pregnant women to reduce the effects of stress. It is therefore important to analyze the associated obstetric and neonatal factors of pregnancy-specific stress in order to improve the well-being of this population^(5,17)^. Research has shown that people with high levels of resilience tend to show fewer depressive symptoms and maintain greater emotional balance^(15,17)^.

Therefore, during pregnancy, which is a period of significant psychosocial adaptation, having a good degree of resilience may prove crucial for coping with the changes inherent in pregnancy and motherhood^(17-20)^. It has been noted that women with high levels of stress tended to have lower levels of resilience, which may also be linked to the development of higher rates of postpartum depression in this group^(5,21,22)^.

Therefore, we established the following research questions: What is the nature and intensity of the relationship between pregnancy-related anxiety, prenatal distress, and individual resilience in pregnant women during the first trimester of pregnancy? Does the obstetric variable of being multiparous influence this relationship?

We feel this research is key to understand the relationship between anxiety, distress, and resilience during pregnancy. In addition, other socio-demographic variables, such as parity or the women’s experience of pregnancy, seem to be determining factors in personalized and quality prenatal care^(3,23,24)^. Early assessment of these variables can help to identify the treatment needs of pregnant women and allow us to plan suitable interventions to promote mental health in this population. Therefore, this study aims to describe and analyze the relationship between pregnancy-related anxiety, prenatal distress, and individual resilience in pregnant women during the first trimester of pregnancy, and compare it with the obstetric variable of parity.

## METHOD

### Study Design

A quantitative, descriptive, cross-sectional study was conducted using non-probability circumstantial sampling.

### Selection Criteria

Pregnant women in the first trimester of gestation belonging to a Regional Management area of the Castilla y León (Spain) Health Service were included. Women with a previous diagnosis of depression, anxiety or psychiatric illness, as well as those with language difficulties were excluded from the study.

### Sample Definition

A total of 360 pregnant women between 11 and 13 weeks of gestation were invited to participate in the study, of whom 144 women eventually completed the questionnaire.

### Data Collection

The sample was recruited in the first trimester of pregnancy at the first obstetric follow-up visit after a 10-minute individual informative session. All the pregnant women who agreed to participate signed the informed consent form and provided their contact details. The questionnaire was then sent by email or WhatsApp® and using the Google Forms® platform, by which the sociodemographic and obstetric variables used in this study (pregnancy-related anxiety, prenatal distress, and resilience) were collected. The average time taken to complete the questionnaire was 10 minutes. In addition, their post-delivery medical records were checked to incorporate neonatal variables (weight, length, and need for resuscitation) into the database. The data collection period was between September 2021 and March 2022 for the questionnaire and between March and October 2022 for the review of the pregnant women’s medical records. Participants did not receive any incentives for participation.

The following instruments were used for the research:

The Prenatal Distress Questionnaire (PDQ)(1), a 12-item scale validated for Spain, which measures pregnancy-specific stress related to maternal concerns, such as medical problems, childbirth, physical symptoms, body changes, and the baby’s health. Responses are given on a 5-point Likert-type scale, where 0 = not at all and 4 = very much. Cronbach’s alpha reliability coefficient is 0.71. Two studies have examined the reliability of the PDQ in high- and low-risk pregnant populations, describing the three categories used in the instrument: “Concerns about childbirth”, “Concerns about relationships” and “Concerns about physical symptoms”(25). The Cronbach’s alpha reliability coefficients for these subscales are 0.77, 0.86, and 0.77, respectively.

The Pregnancy-Related Anxiety Questionnaire (PRAQ-20)^26)^, validated for Spain, which measures five dimensions related to anxiety about being pregnant: concern about changes in themselves, fear for the integrity of the baby, feelings about themselves, fear of childbirth, and worries about the future and their ability as a mother. Each item is scored on a scale from one to five, using a Likert-type scale (5 = strongly agree and 1 = strongly disagree). The reliability of the full PRAQ-20 scale was 0.91 in the first trimester, while the reliability values were 0.78 for concern about changes in oneself and relationships, 0.91 for fear for the baby’s integrity, 0.82 for feelings about oneself, 0.83 for fear of childbirth and 0.71 for concern about the future, all for the first trimester of gestation. The reliability of this scale varies from nulliparous to multiparous women, with 0.92 for nulliparous women in the first trimester and 0.90 for multiparous women. The original authors of the questionnaire set the cut-off point at 67 points.

The Resilience Scale (Resilience Scale-RS-14)(18), validated in Spanish, measures the degree of individual resilience, which is considered a positive personality trait that allows the person to adapt to adverse situations. The scale has an adequate internal consistency (α = 0.79), and levels of resilience are very low for those below 30 points, low between 30 and 48, normal between 49 and 63, high between 64 and 81, and very high for values above 82.

### Data Analysis

A descriptive analysis was performed using measurements of central tendency, dispersion and frequency, with Spearman’s Rho test used to analyze the correlation coefficient and to analyze associations between quantitative variables. The relationship between the quantitative and qualitative variables was determined using the Mann-Whitney U-test, and the Kruskal-Wallis H-test was used to analyze differences between groups. The reliability coefficient (Cronbach’s alpha) was also analyzed. Statistically significant results were established with a p-value <0.05. SPSS v.28 statistical packages were used for data analysis.

### Ethical Aspects

All the participants gave their voluntary informed consent. The protocol was approved by the Ethics Committee of a Spanish University (ETICA-ULE-033-2021) and the Clinical Research Ethics Committee of the Health Areas (Internal Registration No. 21124).

## RESULTS

The sample consisted of 144 pregnant women, with a mean age of 33.57 years (maximum 47 years and minimum 20 years). In terms of parity, 58.3% (n = 84) of the women were multiparous, while 41.7% (n = 60) were in their first pregnancy. Of the total number of women, 90.3% were Spanish, while the remaining 9.7% were foreign. Table 1 describes the sample according to the sociodemographic, obstetric and neonatal variables analyzed.

**Table 1:** Socio-demographic data – Ponferrada, Spain, 2022.

Variable		Mean ± Standard Deviation	n (100%)
Age (years)*		33.57 ± 4.80	
	<35		75 (52.8%)
	≥35		68 (47.2%)
Marital status	Married/cohabiting		123 (85.4%)
	Single/widowed		21 (14.6%)
Nationality	Spanish		130 (90.3%)
	Other		14 (9.7%)
Area of residence	Rural		41 (28.5%)
	Urban		103 (71.5%)
Type of pregnancy	Normal		132 (91.7%)
	Assisted Reproduction		12 (8.3%)
Parity	Primiparous		60 (41.7%)
	Multiparous		84 (58.3%)
Abortions	No		105 (72.9%)
	One or more		39 (27.1%)
Caesareans	No		123 (85.4%)
	One or more		21 (14.6%)
Duration of gestation (weeks)*		38.96 ± 2.31	
Type of delivery	Euthocic birth		72 (50%)
	Dystocic birth		72 (50%)
Epidural	Yes		104 (72.2%)
	No		40 (27.8%)
Episiotomy	Yes		27 (18.8%)
	No		117 (81.3%)
Vaginal tearing	Yes		47 (32.9%)
	No		96 (67.1%)
Weight of newborn (g)*		3138.89 ± 574.73	
Size*		49.24 ± 2.54	
Resuscitation	Yes		17 (11.8%)
	No		127 (88.2%)

Descriptive statistics for the anxiety, stress and resilience variables and their dimensions are shown in Table 2. Participants showed a prevalence of anxiety of 21.5%. In addition, 54.9% reported very high levels of resilience. The mean values and standard deviations obtained for the variables of anxiety, stress and resilience were 55.95 ± 15.32, 18.40 ± 8.40 and 80.28 ± 12.64, respectively.

**Table 2:** Descriptive statistics for pregnancy-related anxiety, antenatal distress and resilience - Ponferrada, Spain, 2022.

Variable/ Sub-variable		n (%)	Min	Max	M	SD	α
Prenatal distress	Total	144 (100%)	0	43	18.40	8.40	0.82
	Concern about childbirth	144 (100%)	0	22	10.42	4.37	0.65
	Relationship concerns	144 (100%)	0	8	3.06	2.34	0.81
	Concern about physical symptoms	144 (100%)	0	12	3.01	2.74	0.68
Pregnancy-related anxiety	Total	144 (100%)	20	100	55.95	15.32	0.91
	Anxiety	31 (21.5%)	67	100	77.16	7.72	
	Without anxiety	113 (78.5%)	20	66	50.13	11.18	
	Concern about changes in oneself	144 (100%)	3	15	6.85	3.29	0.81
	Fear for the baby’s integrity	144 (100%)	7	47	27.42	7.10	0.90
	Feelings about oneself	144 (100%)	3	15	6.89	3.36	0.85
	Fear of childbirth	144 (100%)	4	20	10.36	4.60	0.80
	Concern for the future	144 (100%)	3	15	4.44	2.14	0.75
Resilience	Total	144 (100%)	31	98	80.28	12.64	0.91
	Little	5 (3.4%)	31	47	40.40	6.46	
	Normal	9 (6.3%)	54	62	60	2.69	
	High	51 (35.4%)	64	81	74.06	4.80	
	Very high	79 (54.9%)	82	98	89.13	4.63	

Note: Min: minimum; Max: maximum; M: mean; SD: standard deviation; α: Cronbach’s alpha.

The results of the Mann-Whitney U-test (Table 3) show that primiparous women had higher levels of worry about ­childbirth (PDQ), 11.15 ± 4.58, than multiparous women, 9.90 ± 4.18 (p = 0.05). Primiparous women also had higher levels of anxiety in the fear of childbirth dimension, 12.32 ± 4.81, than multiparous, 8.96 ± 3.91 (p = <0.001). Along the same lines, concern about the future on the PRAQ-20 was higher in the primiparous groups (4.92 ± 2.42 vs. 4.10 ± 1.86, p = 0.007).

**Table 3:** Descriptive statistics of pregnancy-related anxiety, antenatal distress and individual resilience and mean difference by parity – Ponferrada, Spain, 2022.

Questionnaire / Variables		Primiparous		Multiparous		U	p
		M	SD	M	SD		
Prenatal distress	Total	18.88	8.18	1805	8.59	2276.5	0.323
	Concern about childbirth	11.15	4.58	9.90	4.18	2038.5	0.050*
	Relationship concerns	2.70	2.02	3.31	2.53	2209	0.203
	Concern about physical symptoms	3.25	2.78	2.85	2.73	2274	0.314
Pregnancy-related anxiety	Total	57.75	16.93	54.67	14.02	2231.5	0.242
	Concern about changes in oneself	6.78	3.13	6.89	3.43	2514.5	0.982
	Fear for the baby’s integrity	26.83	7.48	27.83	6.83	2279.5	0.328
	Feelings about oneself	6.90	3.52	6.88	3.25	2494	0.915
	Fear of childbirth	12.32	4.81	8.96	3.91	1519.5	<0.001**
	Concerns about the future	4.92	2.42	4.10	1.86	1905	0.007*
Resilience	Total	79.82	12.62	80.61	12.72	2385	0.585

Note: M: mean; SD: standard deviation; U: Mann-Whitney U test; *: p ≤ 0.05; **: p ≤ 0.001.

Significant Spearman’s Rho correlations were found between resilience, anxiety and stress. Thus, high values of resilience correlate significantly with low values of stress (Rho = – 0.412. p < 0.001) and prenatal anxiety (Rho = – 0.370. p < 0.001), and vice versa. On the other hand, the correlation is positive between anxiety and stress (Rho = 0.674. p < 0.001), indicating that women with higher levels of anxiety also have higher stress scores. A positive correlation was also observed between resilience and concerns about physical symptoms, revealing that the greater the resilience, the greater the concern about their physical condition.

The results of the analysis of variance (Kruskal-Wallis) between the groups indicate that there are significant differences between the means of the four levels of resilience (Low. Normal. High and Very High), stress and anxiety. These results are shown in Table 4.

**Table 4:** Comparison between values of resilience and anxiety related to pregnancy and prenatal distress – Ponferrada, Spain, 2022.

Questionnaires/ Variables/ Resilience			M (SD)	Kruskal-Wallis	
				H	p
Prenatal Stress	Total	Little	25.6 (6.07)	18.40	<0.001**
		Normal	26.56 (8.89)		
		High	20.06 (8.75)		
		Very high	15.94 (7.18)		
	Concern about childbirth	Little	4.80 (2.05)	11.73	0.008*
		Normal	8.89 (3.62)		
		High	7.67 (3.13)		
		Very high	6.22 (3.23)		
	Relationship concerns	Little	30.20 (4.76)	7.80	0.050
		Normal	32.33 (4.12)		
		High	28.31 (5.75)		
		Very high	26.10 (7.92)		
	Concern about physical symptoms	Little	9.20 (3.11)	21.18	<0.001**
		Normal	8.89 (3.82)		
		High	7.75 (3.11)		
		Very high	5.96 (3.21)		
Pregnancy-related anxiety	Total	Little	64.20 (11.58)	19.79	<0.001**
		Normal	70.11 (12.49)		
		High	59.35 (12.68)		
		Very high	51.62 (15.82)		
	Concern about changes in oneself	Little	4.80 (2.05)	12.82	0.005*
		Normal	8.89 (3.62)		
		High	7.67 (3.13)		
		Very high	6.22 (3.23)		
	Fear for the baby’s integrity	Little	30.20 (4.76)	8.97	0.030*
		Normal	32.33 (4.12)		
		High	28.31 (5.75)		
		Very high	26.10 (7.92)		
	Concern about changes in oneself	Little	9.20 (3.11)	17.17	0.001**
		Normal	8.89 (3.82)		
		High	7.75 (3.11)		
		Very high	5.96 (3.21)		
	Fear of childbirth	Little	14.80 (5.72)	14.98	0.002*
		Normal	13.56 (3.28)		
		High	11.12 (4.72)		
		Very high	9.23 (4.21)		
	Concerns about the future	Little	5.20 (2.28)	12.12	0.007*
		Normal	6.44 (3.17)		
		High	4.51 (1.69)		
		Very high	4.11 (2.16)		

Note: M: mean; SD: standard deviation; H: Kruskal-Wallis H-test.; *: p ≤ 0.05; **: p ≤ 0.001.

Post-hoc purity tests show that women with very high resilience scores perceive themselves to be less stressed and less worried about childbirth and its physical symptoms than those with normal resilience scores. Similarly, these pregnant women with very high resilience scores report lower values for anxiety about the baby’s integrity, fear of childbirth and worries about the future.

## DISCUSSION

The findings describe the relationship between anxiety, distress and resilience and highlight the importance of considering parity when evaluating mental health in first-trimester pregnant women.

The results suggest that women in the first trimester of pregnancy show a high level of resilience, with the literature showing that a score above 64 points on the RS-14 scale indicates high resilience, and values above 82 indicate very high resilience(18). The average score of the participants in this study was 80.28, with more than 35% of the pregnant women showing high resilience and 54.9% of the population showing very high resilience. Similar studies have found very similar values for the variable of resilience(17).

Global pregnancy-related anxiety was reported by many women, with an average of 55.95 points, and most values below the cut-off point of 67.00 points(26). Only 21.5% of them had anxiety, which is a similar value to that found in another study in a similar population, mostly with primiparous pregnant women in an urban setting, in which 21% of the population had anxiety(27). In this context, the results of this research showed that a greater number of pregnancies were related to the onset of anxiety, which contrasts with other similar research in which data is shown where multiparity acts as a risk factor for anxiety in pregnancy because these women have already experienced a previous pregnancy^(13,28)^.

Regarding the results of the prenatal distress scores, the average score was 18.40 points, which is slightly higher than that found in similar studies using the same instrument^(13,29)^ to study the pregnant population without focusing on a specific trimester. One of these studies presents a PDQ score of 16.87(29), while in the other it is 16.98(13), although in the latter, only 7.1% of the sample belonged to the first trimester of gestation. However, there is another similar study which presents a much higher PDQ score of 23.45 points(14), with, in this case, only 22.3% of the sample belonging to the first trimester of pregnancy.

Pregnancy is perceived as a risk to women’s health and as a danger to the integrity of their future babies(30). Pregnancy-related anxiety and prenatal distress are variables studied in numerous published studies^(27.30)^. In the present research, we observed that primiparous pregnant women show higher values for concerns about the integrity of the baby and have high scores for the dimension of fear of childbirth; these results are similar to those obtained in other similar studies(30). However, in our research, primiparous women experience a higher level of antenatal anxiety and distress during pregnancy than multiparous women.

Resilience may contribute to the well-being of pregnant women and future neonatal and obstetric outcomes. Early assessment of these variables during pregnancy is key, as is promoting resilience from the first trimester. The findings of this research therefore have a significant implication for clinical practice: highly resilient pregnant populations may have better mental health and can cope better with anxiety and stress. More effective health education intervention programs are needed to promote resilience and alleviate or reduce anxiety and distress in pregnant women.

This study has certain limitations which must be considered. Firstly, the lack of representativeness of the sample due to its size, as well as the gestational trimester of all the participants, make it difficult to generalize and extrapolate the results obtained. Secondly, this was a cross-sectional study, and the results obtained do not allow us to conclude the causality between the variables studied. The context of the COVID-19 pandemic must also be taken into account, since, although the date was not collected during the lockdown period, changes in public health policies and socio-economic conditions could have influenced pregnancy rates and other related factors. In addition, reliance on self-reporting and possible limitations in measuring specific variables may have affected data quality. Finally, uncontrolled confounding factors and restrictions in the availability of certain relevant data may have influenced the study’s ability to address specific questions comprehensively.

## CONCLUSIONS

21.5% of the women in the first trimester of pregnancy in a health area in northern Spain had pregnancy-related anxiety and 54.9% had very high levels of individual resilience. Worries about the future and childbirth cause more anxiety and stress in primiparous pregnant women than in those who have already given birth. In addition, pregnant women with high individual resilience show lower values for anxiety and stress than pregnant women with normal resilience. In short, more resilient women show less anxiety and stress during the first trimester of pregnancy.
